# Nanostructured Ternary Metal Tungstate-Based Photocatalysts for Environmental Purification and Solar Water Splitting: A Review

**DOI:** 10.1007/s40820-018-0222-4

**Published:** 2018-10-01

**Authors:** Jun Ke, M. Adnan Younis, Yan Kong, Hongru Zhou, Jie Liu, Lecheng Lei, Yang Hou

**Affiliations:** 1 Key Laboratory of Biomass Chemical Engineering of Ministry of Education, College of Chemical and Biological Engineering, Zhejiang University, 38 Zheda Road, Hangzhou, Zhejiang People’s Republic of China; 2 School of Chemistry and Environmental Engineering, Wuhan Institute of Technology, 693 Xiongchu Ave, Hongshan District, Wuhan, Hubei People’s Republic of China; 3 Department of Environmental Science and Engineering, North China Electric Power University, 619 Yonghua N St, Baoding, Hebei People’s Republic of China

**Keywords:** Ternary metal tungstates, Micro- and nanostructures, Photocatalysis, Environmental purification, Water splitting

## Abstract

Visible-light-responsive ternary metal tungstate (MWO_4_) photocatalysts are being increasingly investigated for energy conversion and environmental purification applications owing to their striking features, including low cost, eco-friendliness, and high stability under acidic and oxidative conditions. However, rapid recombination of photoinduced electron–hole pairs and a narrow light response range to the solar spectrum lead to low photocatalytic activity of MWO_4_-based materials, thus significantly hampering their wide usage in practice. To enable their widespread practical usage, significant efforts have been devoted, by developing new concepts and innovative strategies. In this review, we aim to provide an integrated overview of the fundamentals and recent progress of MWO_4_-based photocatalysts. Furthermore, different strategies, including morphological control, surface modification, heteroatom doping, and heterojunction fabrication, which are employed to promote the photocatalytic activities of MWO_4_-based materials, are systematically summarized and discussed. Finally, existing challenges and a future perspective are also provided to shed light on the development of highly efficient MWO_4_-based photocatalysts.
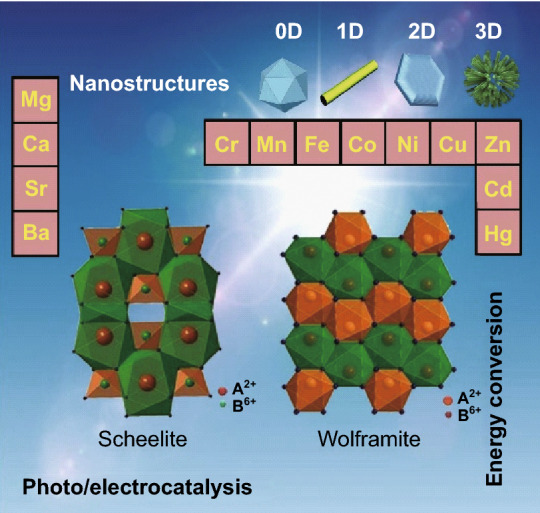

## Highlights

- A series of ternary tungstate-based photocatalysts and their applications in solar energy conversion and environmental purification are systematically introduced.
- The relationship between intrinsic structures and unique properties of ternary tungstate-based photocatalysts is discussed and summarized in detail.
- Various new concepts and innovative strategies are employed to enhance the photocatalytic performance of ternary tungstate-based photocatalysts

## Introduction

**Fig. 1:**
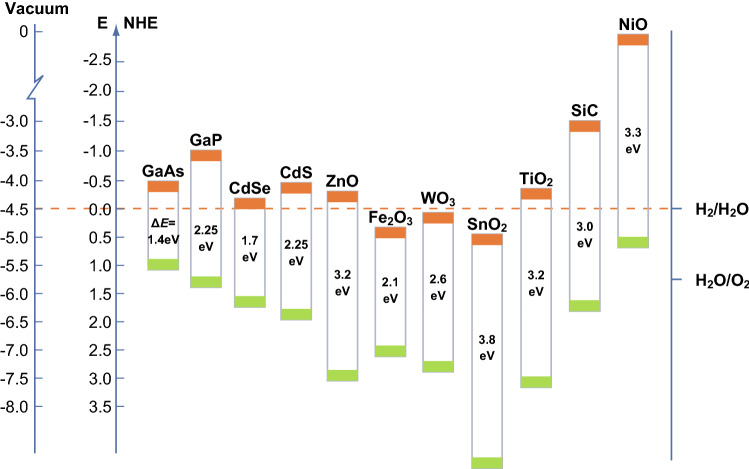
Schematic overview of the band positions of representative semiconductors

Since Fujishima and Honda in 1972 demonstrated that titanium dioxide (TiO_2_) can be used as photoanode to split water excited by ultraviolet light, photocatalysis technology has been viewed as among the most promising approaches to solve the global energy crisis and environmental problems [1–3]. In general, a complete semiconductor photocatalytic cycle involves light-harvesting, photogenerated charge carrier excitation, charge separation and transfer, and surface redox reactions [4–6] that allow for the formation of reactive oxygen species (ROSs), such as free electrons (e^−^), hydrogen peroxide (H_2_O_2_), hydroxyl (·OH), and superoxide radicals (·O_2_^−^) [7, 8]. The aforementioned ROSs play crucial roles in various important applications, including photocatalysis [9–12], photoelectrocatalysis [13–17], plasma photocatalysis [18, 19], and photothermocatalysis [20–22]. Until now, TiO_2_ has been among the most extensively studied semiconductor photocatalysts because of its strong oxidative ability, chemical stability, long durability, and nontoxicity [23–25]. However, the TiO_2_ photocatalyst possesses a wide band gap of ~ 3.2 eV that can only absorb ultraviolet (UV) light, which is a small fraction (~ 5%) of solar light, thereby hardly harvesting the remaining solar energy [26, 27]. To efficiently utilize the majority of the solar spectrum, Fe_2_O_3_- [28], WO_3_- [29, 30], Bi_2_WO_6_- [31], ZnO- [32], Bi_2_O_3_- [33], and NiO-based semiconductors [34] have been widely developed as photocatalysts for environmental treatment and solar water splitting (Fig. 1). Nevertheless, low sunlight utilization efficiency and quantum yield, rapid reverse reactions, and poor stability still hinder practical applications of these photocatalytic materials [35–39].

Desired photocatalysts should have a suitable band gap to achieve a high harvesting efficiency of sunlight, sufficient quantum yield, and an appropriate position of the band edge to trigger redox reactions [40–42]. For an ideal photocatalyst, the conduction band (CB) edge should be sufficiently negative to drive the photo-reduction reaction. In contrast, the valence band (VB) edge should be sufficiently positive to trigger the photo-oxidation reaction [43]. For example, in photocatalytic water splitting, when the VB edge position of the semiconductor photocatalyst is more positive than the potential of H_2_O/O_2_ (1.23 V vs. NHE) and the CB position is more negative than the potential of H_2_/H_2_O (0 V vs. NHE), the water splitting reaction can occur [44–47].

**Fig. 2:**
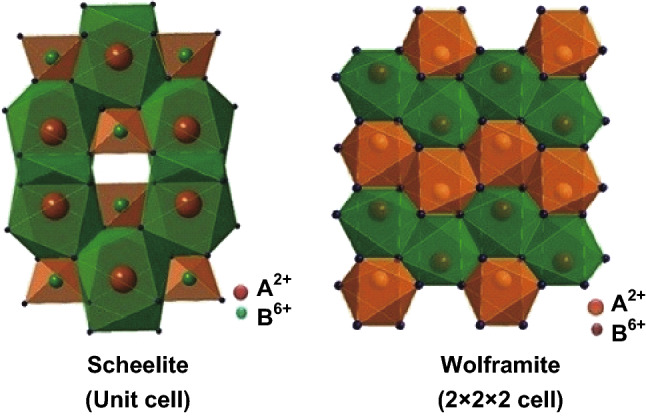
Illustration of unit cells of scheelite and wolframite. Reproduced with permission from Ref. [57]. Copyright 2014 Elsevier

**Fig. 3:**
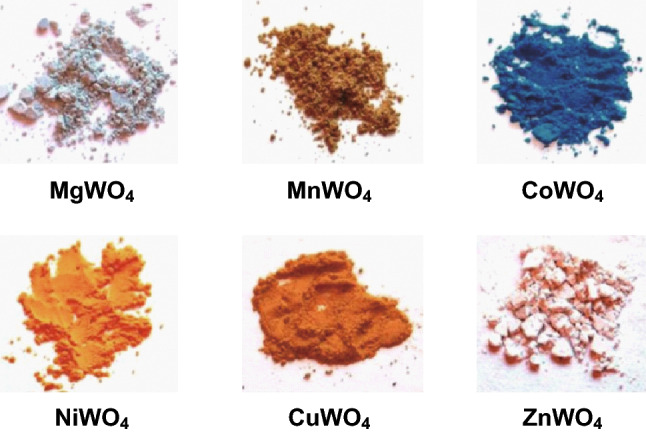
Colors of different MWO_4_ materials. Reproduced with permission from Ref. [51]. Copyright 2014 American Chemical Society

**Fig. 4:**
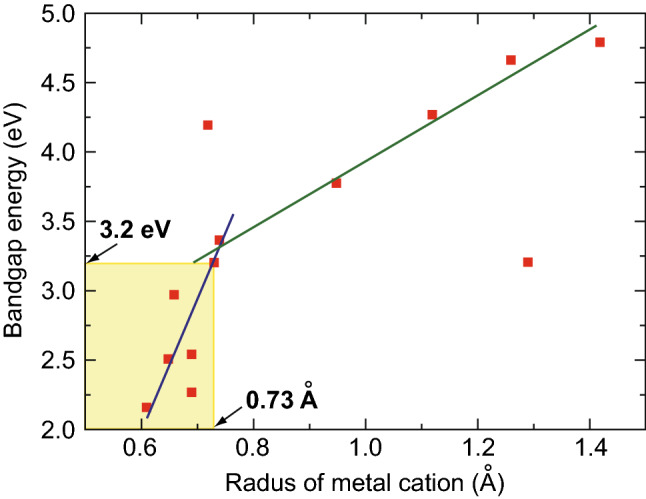
Relationship between band-gap energy of MWO_4_ and the radii of metal cations

Recently, ternary tungstate-based complex oxides have been developed as potential candidates for efficient photocatalytic applications [48–50]. Tungstates are described by the general formula MWO_4_ (M denotes a bivalent cation) [51] and are widely used in the luminescence, microwave ceramics, and catalytic fields, given their self-activating fluorescence effect, microwave, and optical properties [52–55]. Owing to the variety of bivalent cations, the crystal structure of MWO_4_ is dependent on the size of cationic radii. According to the literature [56], MWO_4_ typically has a monoclinic wolframite structure for small M^2+^ cations (M = Fe, Co, Sn, and Ni) and a tetragonal scheelite structure for large M^2+^ cations (M = Ca, Ba, Pb, and Sr), as shown in Fig. 2 [57]. During the past few decades, MWO_4_ with large radii cations, including CaWO_4_ [58, 59], BaWO_4_ [60, 61], PbWO_4_ [62], and SrWO_4_ [63], has been prepared using different synthesis approaches. However, the band gaps of these MWO_4_ photocatalysts are much larger than that of TiO_2_ and are not suitable for practical photocatalytic applications. In contrast, the band gaps of MWO_4_ with small-radii cations are considerably smaller than that of TiO_2_ and could be a promising choice for the efficient utilization of solar energy [64–72]. The band-gap energies of representative MWO_4_ are summarized in Fig. 3 and Table 1. In addition, the band-gap energies of various MWO_4_ and the radii of the metal cations are plotted in Fig. 4. It can be seen that a flock of MWO_4_ is in the yellow area, in which each MWO_4_ has a smaller metal cation radius (< 0.73 Å) and narrower band-gap energy (< 3.2 eV) than those of the others. In contrast, MWO_4_ with a larger cation radius have larger band-gap energy. Notably, for specific MWO_4_, the band gaps presented in Table 1 are not the only ones, because the absorption light range of the semiconductors can be affected and controlled by various factors, including morphology, size, doping, and defects, thus resulting in one semiconductor material possessing several band-gap energies. By comparing the requirements of novel photocatalysts, MWO_4_ with small-radius cations is more advantageous and can be further developed as highly efficient photocatalysts. In addition, it is clearly observed that ternary MWO_4_ systems with a narrow band gap have transition metals as the cation component, which is earth-abundant, cost effective, and low-toxicity, benefiting wide usage in the future. However, for these visible-light-responsive pristine MWO_4_, photocatalytic activities remain inadequate for practical applications because of the rapid recombination of photogenerated holes and electrons.

**Table 1:** Band gaps, crystal sizes, and effective ionic radii of different MWO_4_ materials

Compounds	Ionic radius of cation M (Å)	Band gap *E*_g_ (eV)	Crystalline sizes (nm) via Scherrer equation	References
BaWO_4_	1.42	4.79	55	[56]
PbWO_4_	1.29	3.2	11	[73]
SrWO_4_	1.26	4.66	33	[56]
CaWO_4_	1.12	4.27	32	[56]
CdWO_4_	0.95	3.77	21	[74]
ZnWO_4_	0.74	3.37	32	[75]
CuWO_4_	0.73	3.2	36	[76]
MgWO_4_	0.72	4.19	70	[77]
SnWO_4_	0.69	2.27	36	[78]
NiWO_4_	0.69	2.54	31	[71]
MnWO_4_	0.66	2.97	29	[79]
CoWO_4_	0.65	2.5	35	[80]
FeWO_4_	0.61	2.16	50	[81]

Herein, we provide a comprehensive review of the evolution and current state of the development and application of ternary MWO_4_-based photocatalysts in environmental purification and solar water splitting. First, we discuss the fundamentals of ternary MWO_4_ systems, including the crystal composition, electronic structure, and relationship between the intrinsic structures and properties. Subsequently, versatile reported strategies to improve the photocatalytic activities of pristine MWO_4_ are systematically summarized. Finally, challenges and future developments of ternary MWO_4_-based photocatalysts are discussed. We believe that this review provides information on recent progress in ternary MWO_4_-based photocatalysts for environmental and energy applications and insight into future perspectives, which will aid the design of highly efficient semiconductor-based photocatalysts.

## Ternary MWO_4_ Photocatalysts (M = bivalent metal cations)

The photocatalytic activity of semiconductor photocatalysts is known to be closely related to their crystal and electronic structures [82]. In this section, an overview of the crystal and electronic structures of ternary MWO_4_ is presented and the factors influencing their photocatalytic performance is explored.

### Crystal Structure

**Fig. 5:**
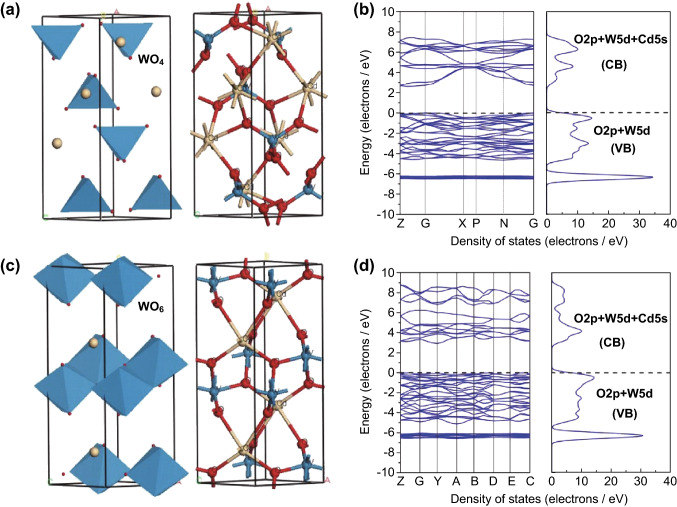
Models of band structures and calculated density of states for tetragonal (**a, b**) and monoclinic (**c, d**) CdWO_4_. Reproduced with permission from Ref. [83]. Copyright 2011 Elsevier

As ABO_4_-type compounds, ternary MWO_4_ complex materials possess a typical wolframite-type monoclinic crystal structure and scheelite-type tetragonal structure. In the scheelite crystal structure, one W atom coordinates with four O atoms to form the WO_4_ tetrahedral unit. In contrast, in the wolframite crystal structure, one W atom is encircled by six oxygen atoms to form the WO_6_ octahedral unit. For example, Yan et al. [83] reported a tetragonal structure in CdWO_4_ material, in which the W atom is situated in the center of the tetrahedra, forming four W–O bonds of the same bond length of 1.758 Å, with the coordination number of the Cd atom being eight. However, the monoclinic structure of CdWO_4_ is similar to that of previously reported MnWO_4_ [84]. Both W(VI) and Cd(II) have octahedral O coordination, in which each octahedron shares two corners with its neighbors. However, the configuration of the WO_6_ octahedron leads to severe distortion in which two W–O bonds are much shorter than the other four W–O bonds. The two crystal phases of CdWO_4_ are shown in Fig. 5. An investigation of methyl orange (MO) degradation showed that the photocatalytic performance of monoclinic CdWO_4_ was much higher than that of tetragonal CdWO_4_ and commercial TiO_2_ under UV light irradiation, which can be ascribed to the lower lattice symmetry of the monoclinic CdWO_4_. Furthermore, the electronic structures of the wolframite- and scheelite-type CdWO_4_ were investigated by theoretical computations and simulations based on density functional theory (DFT). As shown in Fig. 5b-d, both monoclinic and tetragonal CdWO_4_ are indirect-type semiconductors because the calculated bottom of the CB is not situated in the same line as the top of the VB. The calculated band gap of monoclinic CdWO_4_ was larger than that of tetragonal CdWO_4_, which was not consistent with the photocatalytic results. Actually, the monoclinic CdWO_4_ consisted of distorted WO_6_ octahedra, leading to the generation of dipole moments in the WO_6_ octahedral units, while the tetragonal CdWO_4_ comprised normal WO_4_ tetrahedra, forming a highly symmetric lattice in the absence of dipole moments. Based on its crystal and geometric structures, the monoclinic structure of CdWO_4_ is considered a highly efficient photocatalyst, and its catalytic performance has been extensively explored [74, 85, 86].

**Fig. 6:**
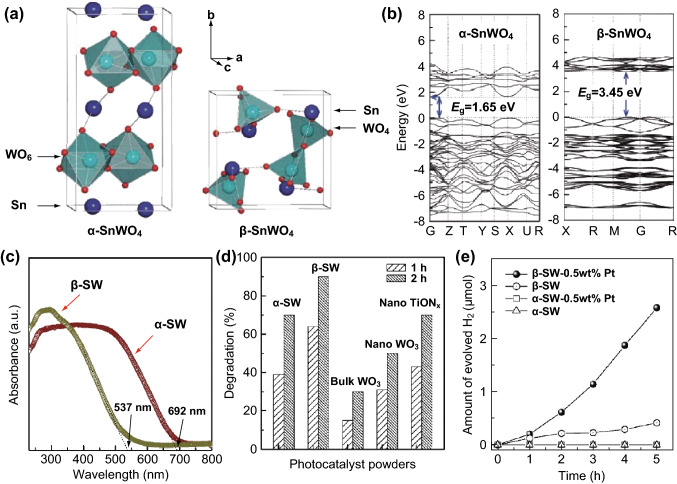
**a** Crystal structures, **b** density of states, and **c** UV–Vis absorption spectra of α-SnWO_4_ and β-SnWO_4_. **d** Photocatalytic activity of α-SnWO_4_ and β-SnWO_4_ in RhB degradation. **e** H_2_ evolution. Reproduced with permission from Ref. [88]. Copyright 2009 American Chemical Society

In addition, it has also been reported that SnWO_4_ has two crystal structures: α-SnWO_4_ and β-SnWO_4_ [87]. The orthorhombic α-SnWO_4_ is comparatively stable in the structure below 670 °C, whereas the cubic β-SnWO_4_ is a steady structure above 670 °C. In the crystal unit of orthorhombic α-SnWO_4_, a single W atom is bonded with six O atoms to constitute typical corner-shared WO_6_ octahedra, while the unshared WO_4_ tetrahedra are composed of a crystal unit of β-SnWO_4_. Owing to the lone-pair effects on the Sn(II) ion, distorted SnO_6_ octahedra are formed in both polymorphs. Cho et al. [88] prepared the aforementioned SnWO_4_ materials with different types of crystal structures and investigated the relationship of the crystal structure with the optical and catalytic properties of SnWO_4_ (Fig. 6). It was demonstrated that a difference in atom arrangement could result in an apparent difference in electronic distribution. The VB of SnWO_4_ is constituted through high hybridization between the Sn 5 s orbital and O 2p orbital, resulting from the strong interaction between the atomic orbitals with closer energy. Meanwhile, the Sn 5 s orbitals contribute to the lower and upper energy levels of the VB, while the O 2p orbitals are dedicated to the middle energy levels of the VB. Therefore, the VB and CB electronic structures of SnWO_4_ are totally different from those of the pristine WO_3_, in which the VB and CB comprise filled O 2p orbitals and empty W 5d orbitals, respectively. The band gap of α-SnWO_4_ was calculated to be 1.65 eV, which was lower than that of WO_3_ (1.77 eV), thereby accounting for the broadening effect of the VB, which can be attributed to the contribution of the Sn 5 s orbitals. In contrast, the calculated band gap of 3.45 eV for β-SnWO_4_ was apparently much larger than that of α-SnWO_4_ and WO_3_. Although the Sn 5 s orbitals also contribute to the VB of β-SnWO_4_ (Fig. 6b), the increase in the band gap stems from the decreased length of the W–O bonds and enhanced crystal field splits, thus resulting in the upshifting of the CB position. Furthermore, the experimental results showed that both α-SnWO_4_ and β-SnWO_4_ exhibited higher photocatalytic performance for the degradation of Rhodamine B (RhB), as compared to other visible-light-response photocatalysts, such as bulk- or nanoWO_3_ and nanoTiON_x_ (Fig. 6d). Moreover, the β-SnWO_4_ showed a higher photocatalytic activity for H_2_ evolution in the presence of methanol, accompanying Pt as a co-catalyst under visible-light irradiation (Fig. 6e), which can be mainly ascribed to the higher CB edge of β-SnWO_4_ compared to that of α-SnWO_4_. Based on the aforementioned analysis, one can conclude that the MWO_4_ photocatalysts usually possess more than two types of crystal structure, thus leading to a difference in geometries and local lattice distortions. The non-bonded electrons in the metal ion should be considered an important factor in analyzing the crystal field, particularly in an MWO_4_-based asymmetric coordination environment. The distortion of the local crystal structure influences the electronic structure and band distribution, thus affecting the catalytic performances of MWO_4_-based photocatalysts.

### Electronic Structure

Generally, tungstates are considered derivatives of H_2_WO_4_ and WO_3_ because of their similar elemental constitutions and crystal structures [89–91]. The DFT [92] and ab initio [93] calculations indicate that the CB of MWO_4_ consists of W 5d orbitals in tungstates, as in WO_3_, while the O 2p orbitals only comprise the upper part of the VB because the bivalent metal cation in tungstates contributes differently to the VB and CB positions given different outer electronic arrangements [55].

**Fig. 7:**
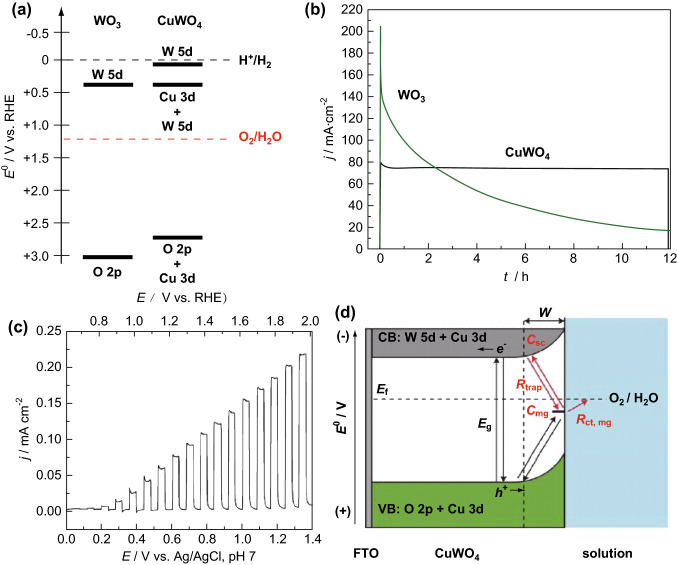
**a** Distribution of energy level and **b** chronoamperometry curves of CuWO_4_ and WO_3_. **c** Polarization curve of CuWO_4_ photoanode under AM1.5G irradiation. **d** Illustration of the physical model of charge carrier transfer in CuWO_4_. Reproduced with permission from Ref. [102]. Copyright 2016 American Chemical Society

For instance, visible-light-driven CuWO_4_ is employed for photoelectrochemical (PEC) water splitting because of its suitable band gap of 2.3 eV [94–96]. For most binary metal oxides, the VB is mainly constituted of O 2p orbitals; thus, the VB energy is usually in the range of 2.5–3.0 eV, indicating that the top of the VB is not significantly shifted in the metal oxides. For ternary metal oxides, however, the mixing atomic orbitals between the O 2p orbitals and metal orbitals could result in an apparent shift in the top position of the VB [97]. Thus, for CuWO_4_, the VB shifts upward, in comparison to that of WO_3_, accounting for the hybridization between the O 2p orbitals and Cu 3d orbitals (Fig. 7a). The upward movement in the VB position indicates a decrease in the band gap, which results in an increased visible-light absorption range [98]. However, the composition of the CB is still a topic of hot debate. DFT calculations show that the VB of CuWO_4_ is composed of O 2p orbitals with a small portion of Cu 3d, whereas the CB of CuWO_4_ is composed of W 5d orbitals [99, 100]. Moreover, the Cu 3d orbitals may contribute to the CB of CuWO_4_ except for the top of the VB [101]. Experimental results demonstrate that the CB shift of CuWO_4_ relative to that of WO_3_ can be ascribed to the incorporation of Cu 3d orbitals into the energy level of the CB [102]. Yet, strong evidence of an accurate contribution proportion of Cu 3d orbitals to the CB of CuWO_4_ has not been obtained. Although the CB composition of CuWO_4_ is unclear, the CuWO_4_ photoanode presented a high photocatalytic activity with a photocurrent density of up to 0.07 mA cm^−2^ at 1.23 V and a high stability under AM 1.5G illumination (Fig. 7b, c), indicating that the CuWO_4_ photoanode has a thermodynamic potential for water oxidation. In this system, a physical model of the photogenerated charge carrier pathways in CuWO_4_ is proposed as shown in Fig. 7d. It shows that when the photogenerated electrons are transferred from a solution medium to a mid-gap state, the thermodynamic potential of the mid-gap state can be utilized to determine which elementary reaction is favored to occur and which is the rate-limiting reaction.

Apart from CuWO_4_, the electronic structures of other MWO_4_ materials have also been previously studied. For example, Rajagopal and co-workers [103, 104] used X-ray emission spectroscopy and DFT computation to study the electronic structures and related properties of FeWO_4_ and CoWO_4_ photocatalysts. The theoretical calculation results demonstrated that O 2p orbitals mainly contributed to the VB of tungstates, while the unoccupied Fe 3d orbitals and Co 3d orbitals were dominantly dedicated to the CB of FeWO_4_ and CoWO_4_, respectively. In addition, the density of states showed that Co 3d and W 5d orbitals also contributed to the VB regions of CoWO_4_, similar to that of FeWO_4_. Hence, the VBs of FeWO_4_ and CoWO_4_ are composed of O 2p, W 5d, and Fe/Co 3d orbitals. However, X-ray emission spectroscopy results revealed that the W 5d orbitals and O 2p orbitals are dedicated to the entire VB of the tungstates, in which the O 2p orbitals are dedicated to the upper region of the VB and W 5d orbitals are dedicated to the lower region of the VB. The theoretical results agreed well with the experimental results for FeWO_4_ and CoWO_4_. In addition, the electronic structure of NiWO_4_ was obtained. For NiWO_4_, the CB predominantly consists of W 5d orbitals and Ni 3d orbitals, while the VB primarily consists of Ni 3d orbitals and O 2p orbitals [105]. It was found that the VB composition of NiWO_4_ is different from those of FeWO_4_ and CoWO_4_, which is related to the energy level distribution of the orbital electrons around the metal ions. Therefore, based on the aforementioned results, one can conclude that the electronic structures of ternary MWO_4_ systems mainly depend on the position of the M^2+^ cation in the periodic table, which affects the outer electronic arrangements and hybridized electrons of the atomic orbital to the M^2+^ cation.

## Strategies for Enhanced Photocatalytic Activity

As described in Sect. 1, ternary MWO_4_ systems can act as highly promising photocatalysts for environmental purification and solar water splitting. However, among the major limiting factors in enhancing their photocatalytic activities is the rapid recombination of photogenerated electron and hole pairs. To overcome this problem and improve the overall catalytic performance of MWO_4_ photocatalysts, many research groups have endeavored to develop various techniques to enhance the separation and transfer efficiency of photoexcited charge carriers inside MWO_4_ or at the interface between different components. In this section, an overview of the developed strategies is provided to offer insights on their effects for the separation efficiency of photogenerated charge carriers and the corresponding catalytic performance of MWO_4_-based photocatalysts.

### Morphological Control

**Fig. 8:**
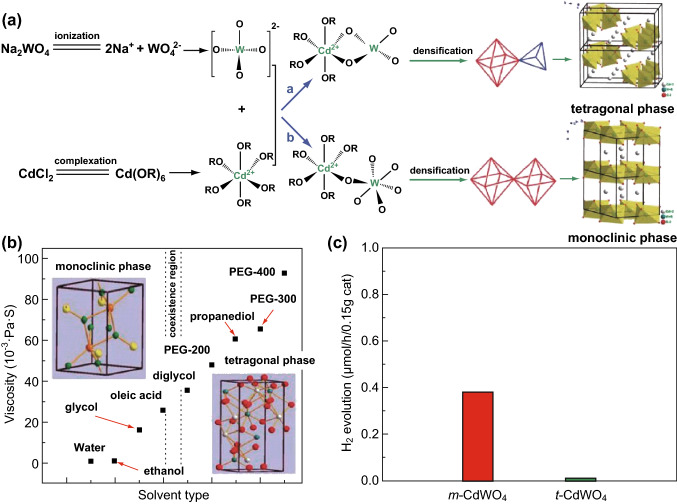
**a** Formation mechanism of m-CdWO_4_ and t-CdWO_4_ and the **b** relationship between different crystal phases of CdWO_4_ and used solvents. **c** Photocatalytic H_2_ evolution of m-CdWO_4_ and t-CdWO_4_ under visible light irradiation. Reproduced with permission from Ref. [86]. Copyright 2012 Royal Society of Chemistry

As is known, the morphology, facet exposure, and dimensions of photocatalysts have a significant influence on the photocatalytic performance. For example, nanostructured photocatalysts can exhibit excellent photocatalytic activities because the morphologies and particle sizes of the photocatalysts have a significant influence on the optical and electronic properties, which in turn affect the photocatalytic activities. For example, Yu et al. [106] prepared FeWO_4_ samples with different morphologies, including nanoparticles, flakes, nanorods (NRs), and a mixture of NRs with flakes and tiny granules, by varying pH values during the hydrothermal process, and systematically investigated their optical properties. The results indicated that the band-gap values of FeWO_4_ are correlated with specific morphologies. Hosseinpour-Mashkani and co-workers [75] synthesized ZnWO_4_ nanoparticles with different sizes through a precipitation route using different polymeric surfactants. The photocatalytic degradation experiments of MO demonstrated that the ZnWO_4_ with the smallest size of 27 nm exhibited the highest photoactivity, compared with other ZnWO_4_ samples under UV light illumination. It has been demonstrated that particle size can affect the band gap of semiconductors because of quantum size effects. The exposed surface area of nanoparticles increases with a decrease in nanoparticle size, which can provide more active sites for a surface catalytic reaction. In addition to morphologies and particle sizes, size-related crystallinity of MWO_4_ has an important influence on the photophysical and photocatalytic properties. For example, Tong et al. [74] reported nanostructured CdWO_4_ with controllable particle sizes via a hydrothermal process and studied the effects of the particle sizes on the lattice symmetry and crystallinity. It was found that the decreased size of the CdWO_4_ nanoparticles resulted in an expanded lattice, lowered crystallinity, and broadened band gap. Meanwhile, the decrease in particle sizes caused an apparent decrease in the photocatalytic activity of CdWO_4_. These results illustrate that size-related properties are closely correlated with the photocatalytic activity of MWO_4_. Apart from the effects of the aforementioned factors on photoactivity, the crystal phase of MWO_4_ is also regulated by various synthetic routes to obtain the desired physiochemical and optical properties, because different crystal phases can lead to distinct differences in the exposure of crystal facets and reactivities. For instant, different crystal phases of CdWO_4_ nanocrystals were prepared by adjusting the used solvents for photocatalytic H_2_ evolution (Fig. 8) [86]. The solvent significantly affected the chelation and growth of the CdWO_4_ material, thus leading to apparent differences in the crystal phase. m-CdWO_4_ nanocrystals with particle sizes ranging from 4.4 to 31 nm can be prepared using short-chain solvents, while t-CdWO_4_ nanocrystals can be easily prepared utilizing long-chain solvents. The obtained m-CdWO_4_ nanocrystals exhibited much higher H_2_ production than the t-CdWO_4_ nanocrystals.

**Fig. 9:**
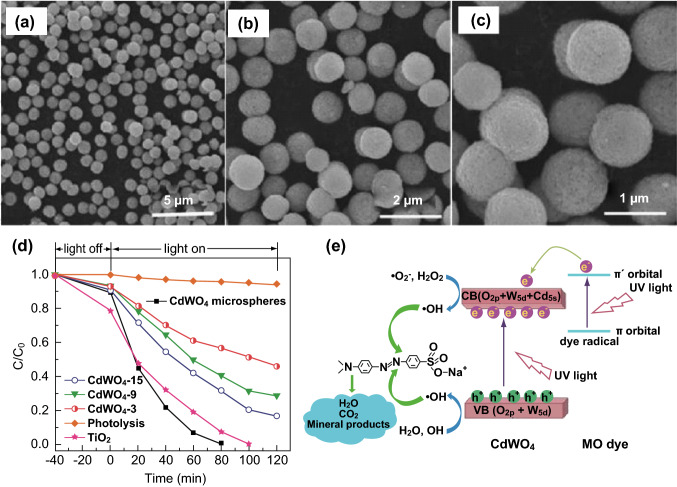
**a–c** FESEM images of CdWO_4_ microspheres. **d** Photocatalytic degradation efficiencies of MO in the presence of different photocatalysts. **e** Photocatalytic mechanism for CdWO_4_ microspheres. Reproduced with permission from Ref. [107]. Copyright 2014 Royal Society of Chemistry

**Fig. 10:**
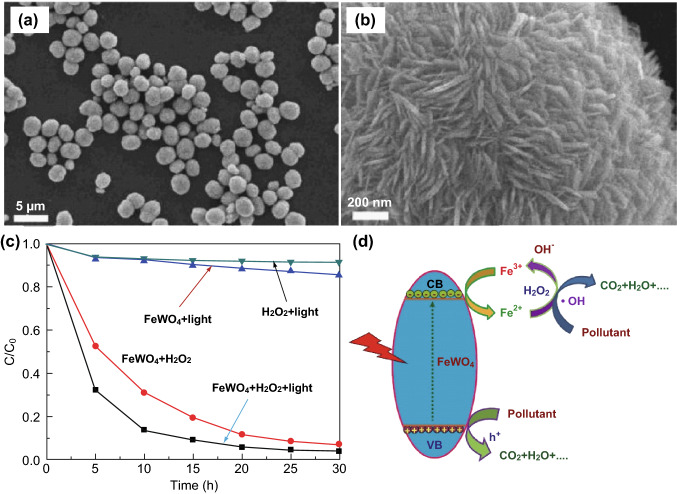
**a**, **b** FESEM images of FeWO_4_ microspheres. **c** Photocatalytic performance of FeWO_4_ microspheres for the degradation of RhB. **d** Schematic illustration of photocatalysis mechanism for FeWO_4_ microspheres. Reproduced with permission from Ref. [112]. Copyright 2016 Elsevier

During recent years, the construction of hierarchical structures to tune the morphologies of MWO_4_ photocatalysts has attracted more attention, because hierarchical structures can offer more exposed surfaces and/or active sites. For example, highly efficient hydrophobic CdWO_4_ microspheres were synthesized by Hou et al. [107] through a microwave-assisted interfacial hydrothermal strategy. The CdWO_4_ microspheres showed enhanced photocatalytic activity for the degradation of MO under mercury lamp irradiation (Fig. 9). The advantages of a hierarchical structure for the enhanced photocatalytic activity of MWO_4_ are supported by several research groups [108–111]. Chen et al. [112] prepared FeWO_4_ microspheres by using an ethylene glycol-assisted solvothermal approach, in which ionic1-octyl-3-methylimidazolium tetrachloroferrate was used as one of the starting materials and played an important role as both a reactant and template. The microsphere-like FeWO_4_ material consisted of numerous nanosheets and exhibited a much better photo-Fenton activity in water purification (Fig. 10) because of the generation of hydroxyl radicals, which are produced from the chemical reaction between Fe^2+^ on the surface of FeWO_4_ and H_2_O_2_. The formed Fe^3+^ was further reduced by the photoinduced electrons to generate Fe^2+^, which is a virtuous cycle, to maintain high photocatalytic performance. Recently, Zhou et al. [113] synthesized an urchin-like MnWO_4_ hierarchical structure through a facile hydrothermal process with the assistance of cetyltrimethylammonium bromide (CTAB) as surfactant. The introduction of CTAB as the surfactant had significant effects on the morphology and magnetic properties of the MnWO_4_ nanocrystals. Subsequently, Xing et al. [114] provided a new route for the synthesis of a complex three-dimensional (3D) MnWO_4_ nanostructure. In this case, a 3D flower-like MnWO_4_ nanocomposite was synthesized using a microemulsion-based solvothermal approach. Moreover, Chen et al. [115] discussed the effect of the morphology of β-SnWO_4_ on its photocatalytic performance, in which the β-SnWO_4_ with a multi-armed architecture and hexahedral symmetry displayed much higher photocatalytic activities than those of both cubic β-SnWO_4_ and commercial WO_3_. Hence, more surface reaction sites induced by the hierarchically multi-armed architecture and the band structure reframing caused by incorporation of the Sn atom into WO_3_ contributed to the excellent photocatalytic activity.

**Fig. 11:**
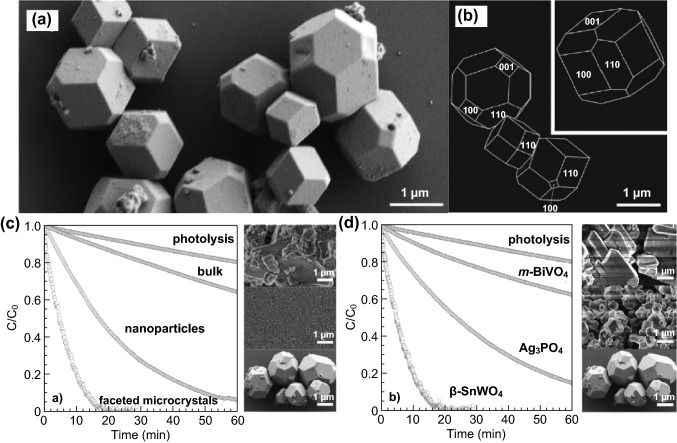
**a**, **b** FESEM images of faceted β-SnWO_4_ microcrystals. **c, d** FESEM images and photocatalytic activities for the degradation of MB over faceted β-SnWO_4_ microcrystals, bulk β-SnWO_4_, and β-SnWO_4_ nanoparticles under sunlight irradiation. Reproduced with permission from Ref. [117]. Copyright 2012 Royal Society of Chemistry

Previously, crystal facet exposure was viewed as an effective strategy to regulate the surface physicochemical and photophysical properties, thus optimizing the reactivity and selectivity of photocatalysts. In general, a crystal facet with a high percentage of unbonded atoms has superior reactivity in comparison to that with a low ratio of unpaired atoms. In addition, crystal facets possessing high surface energy are usually unstable during preparation. It is desirable to synthesize photocatalysts with a high exposure of high-energy crystal facets to enhance the photocatalytic reactivity and selectivity. Using DFT calculations, Qiu and co-workers [116] reported the atom distributions and electronic properties of MnWO_4_ and FeWO_4_ with specific facets. The calculated results showed that the {010} and {100} facets have the lowest surface energies in wolframite-type FeWO_4_ and MnWO_4_, respectively. Meanwhile, it was observed that the Fe and Mn atoms on the {010} and {001} planes as absorption sites can be used to absorb anions. These results indicated that the exposed {100} facet in MnWO_4_ and FeWO_4_ can provide a path for improving their photoactivities, while the other exposed facets can offer a certain selectivity to a specific reaction, such as a dichlorination reaction. Recently, Ungelenk et al. [117] synthesized phase-pure β-SnWO_4_ with truncated rhombic dodecahedrons using a microemulsion technique with CTAB as a co-surfactant with n-hexanol (Fig. 11). Benefitting from the rapid Na_2_WO_4_-induced nucleation and slow crystal growth controlled by the CTAB-mediated microemulsion reaction, the as-prepared β-SnWO_4_ with truncated rhombic dodecahedrons was encircled with highly exposed {100} and {110} facets. By comparing a series of β-SnWO_4_ to other photocatalysts of Ag_3_PO_4_ and m-BiVO_4_, the β-SnWO_4_ microcrystals with exposed {100} and {110} facets showed outstanding photocatalytic activity for the degradation of organic pollutants under daylight irradiation, which was far better than that of other photocatalysts, including bulk non-faceted β-SnWO_4_ and spherical-like β-SnWO_4_ nanoparticles. In addition, given the slight difference in specific surface area between the faceted β-SnWO_4_ and non-faceted β-SnWO_4_, it was concluded that optimized facet exposure was the predominant reason for the distinct photocatalytic activity. In addition, Tian et al. [118] reported a facile solvothermal method for the synthesis of hierarchical FeWO_4_ nanosheets with an exposed {100} facet, which exhibited excellent peroxidase-like catalytic activity for oxidizing the peroxidase substrate of 3,3′,5,5′-tetramethylbenzidine (TMB) because of the formation of hydroxyl radicals in the presence of H_2_O_2_. The surface Fe^2+^ in FeWO_4_ can activate the H_2_O_2_ molecule to produce active hydroxyl radicals for the oxidation of TMB. The results clearly indicated that the {100} facet of FeWO_4_ had a much higher ratio of Fe atoms than that of the {001} and {010} facets, which explained the enhanced catalytic activity of FeWO_4_ with the exposed {100} facet.

Owing to the high surface area and large absorption cross section it provides, a low-dimensional nanostructure can be constructed to manipulate and regulate optical, electrical, and magnetic properties [119, 120]. Low-dimensional nanostructures including those that are one-dimensional (1D) or two-dimensional (2D) cause the growth of a crystal along one or two directions, which can contribute to more exposure of specific surface facets. For example, 1D CdWO_4_ NRs were prepared using microwave or hydrothermal approaches and exhibited excellent photocatalytic activity for environmental treatments, as compared to nanoparticles [121–123]. Kovacs and co-workers [111] prepared a series of FeWO_4_ with different morphologies, including nanoparticles, NRs, and nanosheets, by varying the Fe precursors. The nanosheet-like FeWO_4_ with band-gap energy of ~ 2.2 eV exhibited excellent absorption ability throughout the UV and visible-light range, which was attributed to the formation of a cavity assembled by nanosheets that resulted in enhanced photon harvest. Therefore, the FeWO_4_ nanosheets showed higher photocatalytic activity than other control samples for the degradation of organic dyes under visible-light irradiation.

### Surface Modification

Considering that the photocatalytic reaction proceeds on the surface of semiconductors, the surface physiochemical properties of semiconductor-based photocatalysts are important for improvement of photocatalytic activity. Several strategies, including chemical etching, surface coverage, and co-catalyst attachment, have been developed to tune the surface properties of semiconductor-based photocatalysts.

The purpose of etching techniques, such as chemical etching and laser or electron-beam irradiation, is to form non-stoichiometric or metal/oxygen vacancies on the surface of an inorganic semiconductor. The formation of metal or oxygen vacancies has proven to have an apparent influence on the electronic distribution because of the introduction of a new defect energy level, thus affecting the light absorption range and photocatalytic activity. For example, Aloysius-Sabu et al. [124] investigated the effects of intentional electron-beam irradiation on the crystal phase, size, and surface properties of CaWO_4_ that was prepared through chemical precipitation and heat treatment. The experimental results showed that the high-energy electron beam significantly affected the crystal size and surface structure, but not the crystal phase. When the CaWO_4_ photocatalyst was irradiated by an electron beam, the surface atomic layers of CaWO_4_ underwent stretching and compressive strain, which resulted in the formation of surface defects and a new energy level in the band gap. Therefore, an apparent absorption tail and narrowed band-gap energy were observed in the irradiated CaWO_4_ sample. In addition, Lin et al. [125] prepared a visible-light-driven Ag_2_WO_4_ photocatalyst through a laser irradiation route in liquid using commercial Ag_2_WO_4_ as a starting material for organics degradation and H_2_ evolution. Because of the laser irradiation, the crystal structure was recrystallized and a lattice defect was introduced in Ag_2_WO_4_, leading to the formation of intermediate energy levels with a decrease of 0.44 eV in the band gap. The synthesized Ag_2_WO_4_ exhibited a photocatalytic H_2_ evolution rate as high as 13.73 μmol (hg)^−1^ under visible-light illumination, while no H_2_ evolution was observed in the unirradiated commercial Ag_2_WO_4_ sample, which was ascribed to a large band gap of 3.22 eV for bulk Ag_2_WO_4_.

However, to enhance the solar conversion efficiency of tungstates, surface coverage has been adopted to increase the charge transfer efficiency by means of passivating the surface via deposition of a protective layer. For example, Karthiga and co-workers [71] reported the synthesis of NiWO_4_ nanoparticles modified by a plant extract, *A. indica*, as a capping agent through a precipitation route for enhanced photocatalytic activity. The introduction of *A. indica,* which possesses rich water-soluble heterocyclic groups, led to the formation of a passivation layer on the surface of the NiWO_4_ nanoparticles, which allowed the NiWO_4_ nanoparticles to separate well from each other. In comparison to the bare NiWO_4_ nanoparticles, the *A. indica*-coated NiWO_4_ exhibited a much higher photocatalytic activity for the degradation of organic contaminants under visible-light irradiation because of the formation of the passivation layer of the plant extract, which significantly suppressed the recombination of photoinduced electrons and holes. Meanwhile, modified NiWO_4_ presented higher antimicrobial activity as compared with pure NiWO_4_ nanoparticles.

**Fig. 12:**
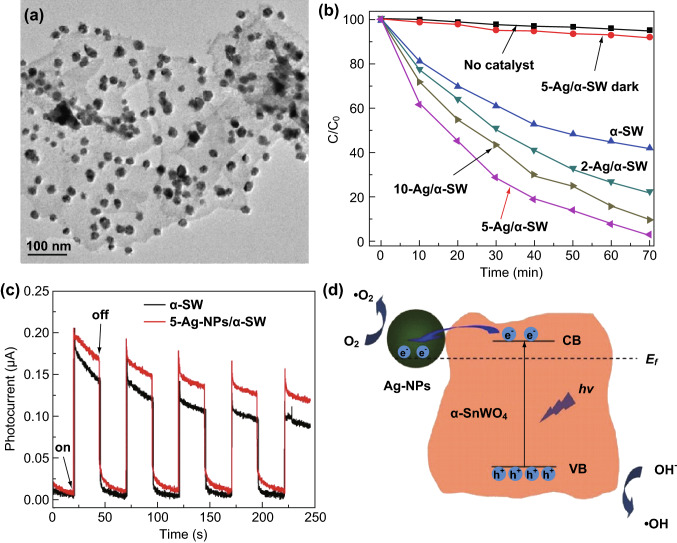
**a** TEM image and **b** photodegradation efficiency of Ag NPs/α-SnWO_4_ under visible-light irradiation. **c** Transient photocurrent response for different samples. **d** Illustration of photocatalytic mechanism of Ag NPs/α-SnWO_4_. Reproduced with permission from Ref. [130]. Copyright 2017 Elsevier

Apart from chemical etching and surface coverage strategies, attachment of noble metal co-catalysts (such as, Pt, Au, and Ag) is another effective means to tune the surface photophysical properties of photocatalysts because of the surface plasmon resonance (SPR) effect [126, 127]. The SPR effect not only significantly enhances visible-light absorption, but also produces a localized electromagnetic field, thus improving the separation efficiency of the photogenerated charge carriers at the interfaces between the metal and semiconductor [128, 129]. Furthermore, the metal–semiconductor heterostructure could efficiently suppress the recombination of photogenerated electrons and holes because of the formation of Schottky barriers at the contacted interface, thus enhancing photocatalytic performance [126]. Based on the aforementioned features, the introduction of a noble metal into MWO_4_ is a feasible approach to realize enhancement of its photocatalytic performance. Recently, Liu et al. [130] prepared Ag nanoparticles (NPs)/α-SnWO_4_ nanosheets through microwave-assisted anchoring of Ag NPs on SnWO_4_ nanosheets. The loading amount of the deposited Ag NPs was well-tuned by adjusting the initial concentration of the Ag^+^ precursor and CTAB surfactant (Fig. 12a). The obtained Ag NPs/α-SnWO_4_ hybrid showed enhanced light absorption ability and photocatalytic activity for the degradation of MO, compared to pure α-SnWO_4_, under visible-light irradiation. Moreover, the hybridized Ag NPs/α-SnWO_4_ system exhibited improved transient photocurrent density in comparison to that of pristine α-SnWO_4_ (Fig. 12c), indicating that more photoinduced charge carriers could be produced and further participated in the redox reaction. In addition, Yan et al. [131] synthesized Ag-loaded CdWO_4_ NRs using a photo-assisted co-precipitation method with the addition of the PEG-100 surfactant, which exhibited a higher photocatalytic activity than that of pure CdWO_4_ NRs because of the SPR effect. In another study, Au NPs were utilized to construct Schottky barriers in an MWO_4_-based system. For instance, Au NRs/MnWO_4_, with a diameter of 20-40 nm, was reported by Chakraborty et al. [132] to enhance the photocatalytic decomposition of 2-propanol and phenol. The Au NPs as co-catalysts in the Au/MnWO_4_ hybrid were beneficial to multi-electron O_2_ reduction and hole oxidation.

### Heteroatom Doping

It has been demonstrated that the introduction of impurities via doping of heteroatoms into a semiconductor can influence the photocatalytic performance [133, 134]. However, heteroatom doping might either have positive or negative impacts for the photocatalytic performance of semiconductors, because there are two different doping levels—the shallow level near the surface and deep level inside the body [135]. Deep-level doping can usually provide a recombination center to intensify the meaningless dissipation of absorbed photons, thus undermining the photocatalytic activity. Therefore, appropriate heteroatom doping is vital to increasing the photocatalytic performance of heteroatom-doped photocatalysts. To overcome shortcomings, such as a narrow wavelength range and rapid recombination of photogenerated electron–hole pairs, a few recent reported types of heteroatom doping to enhance the photocatalytic performances of ternary MWO_4_ materials are reviewed and their roles discussed in detail.

**Fig. 13:**
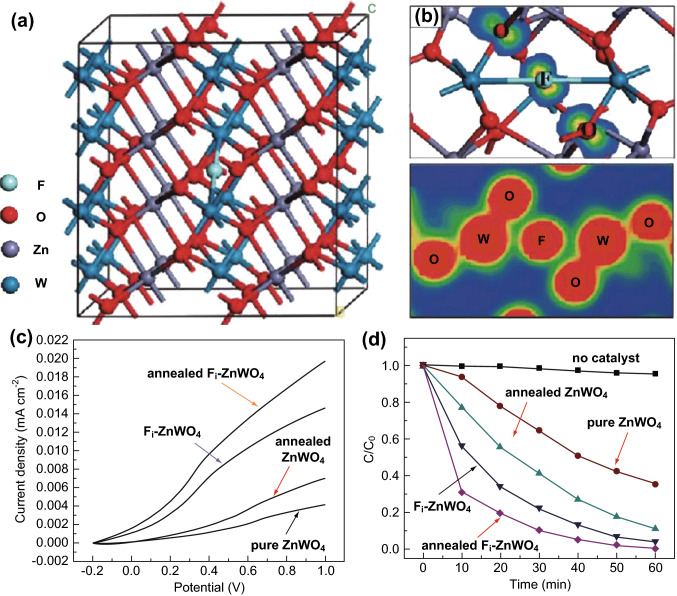
**a** Geometric structures for the F_i_-ZnWO_4_. Spin density (top) and total charge density (bottom) for F_i_-ZnWO_4_. **c** Photocurrent density of the synthesized samples under UV-light irradiation. Photocatalytic degradation of RhB over different samples under UV light irradiation. Reprinted with permission from Ref. [140]. Copyright 2010 American Chemical Society

In heteroatom doping, the dopants are mainly non-metal elements such as B [136], Cl [137], and various transition metals (Zn, Ni, Fe, Co, etc.) [138, 139], which exhibit enhanced photocatalytic activity for mineralizing organic pollutants. For instance, Chen et al. [140] synthesized F-doped ZnWO_4_ nanocrystals (F_i_-ZnWO_4_ nanocrystals) using a two-step hydrothermal process and investigated its chemical bonds via band structure calculations (Fig. 13). In comparison to undoped ZnWO_4_, the F_i_-ZnWO_4_ nanocrystals exhibited significant red shifts and improved light absorption in the range of UV–visible light, which resulted in enhancement of photocatalytic activity for the degradation of RhB under mercury lamp irradiation. Additionally, the experimental results showed that the crystallinity and morphology of the prepared F_i_-ZnWO_4_ was strongly related to the photocatalytic activity. F_i_-ZnWO_4_ exhibited higher photocatalytic performance for the degradation of organic dyes than F_i_-ZnWO_4_ NPs. Based on the theoretical calculation results, the enhancement of the photocatalytic activity of F_i_-ZnWO_4_ might be ascribed to the following reasons. First, F-doping increased the absorptivity of F_i_-ZnWO_4_, such that it could absorb more reactants to enhance the photocatalytic activity. Second, a new half-filled state was introduced into the original band gap of ZnWO_4_ accompanying the F-doping, which could provide more holes to enhance the photocurrent density of F_i_-ZnWO_4_. Thus, heteroatom doping could efficiently improve the photocatalytic activities of MWO_4_ by introducing a new energy level to regulate the original electronic structure.

Apart from the introduction of non-metal elements, transition metal elements can also be a potential dopant. For example, Su et al. [141] prepared Zn^2+^-doped SnWO_4_ nanocrystals, and reported that the morphological alteration of SnWO_4_ nanocrystals from nanosheets to nanowires can be controlled by Zn^2+^ doping. Consequently, the Zn^2+^-doped SnWO_4_ exhibited a greater Brunauer–Emmett–Teller surface area (54 and 100 m^2^ g^−1^ for pure SnWO_4_ and Zn^2+^-doped SnWO_4_, respectively), narrowed band gap (2.68 and 2.64 eV for pure SnWO_4_ and Zn^2+^-doped SnWO_4_, respectively), and highly enhanced photocatalytic performance for the degradation of MO (95% and 30% for pure SnWO_4_ and Zn^2+^-doped SnWO_4_, respectively) compared to that of pure SnWO_4_. In addition, Song et al. [142] reported the synthesis of Zn-doped CdWO_4_ NRs using a hydrothermal process to enhance the photocatalytic conversion efficiency of organic pollutants into low-toxicity small molecules under simulated sunlight irradiation. The influence of the Zn-doping amounts on the crystal phase, morphology, and optoelectronic properties of CdWO_4_ NRs was also systematically investigated. Compared to the undoped sample, the Zn-doped CdWO_4_ NRs exhibited much higher photocatalytic activity, which was assigned to the narrowed band gap due to Zn-doping. Heteroatom doping could be an effective means to tune the distribution of the energy level and further enhance the photocatalytic performance of MWO_4_ without consuming excess foreign substances.

Recently, rare earth element-doped photocatalysts have attracted more attention because of their special 4f electron configuration, which could be beneficial for introducing a suitable energy level into the original band gap of MWO_4_ [143]. Phuruangrat et al. [144] synthesized Ce-doped ZnWO_4_ using a hydrothermal process and investigated the influence of Ce doping on the crystal phase, morphology, electronic structure, and photocatalytic activity of ZnWO_4_. After the introduction of Ce atoms, the photocatalytic activity of ZnWO_4_ improved with the increase in Ce content, owing to the following two reasons. First, the introduction of the Ce^3+^ dopant led to the generation of defects on the surface of Ce-doped ZnWO_4_. Second, the Ce^4+^ ions on the surface of ZnWO_4_ could efficiently trap electrons at the CB by the reduction of Ce^4+^ into Ce^3+^ ions, thus efficiently suppressing the recombination of photoinduced electrons and holes in the Ce-doped ZnWO_4_. Therefore, rare earth elements with variable valence, such as Ce, La, and Eu, are promising dopants to improve the photocatalytic activity by trapping photogenerated electrons, consequently limiting the recombination of photogenerated charge carriers.

### Heterojunction Fabrication

#### Hybridization with Semiconductors

Among the aforementioned approaches, constructing a semiconductor heterostructure is an effective means to obtain highly efficient photocatalysts [145–148]. When heterojunctions are composed of different semiconductors that have matching band potentials to form type-I or type-II heterojunctions through realignment of the energy level, the excited photogenerated holes and electrons can be moved from one semiconductor to another in opposite directions driven by the formed built-in electric fields [149, 150], thus strengthening the separation efficiency of the photoinduced electrons and holes and further enhancing photocatalytic performance.

**Fig. 14:**
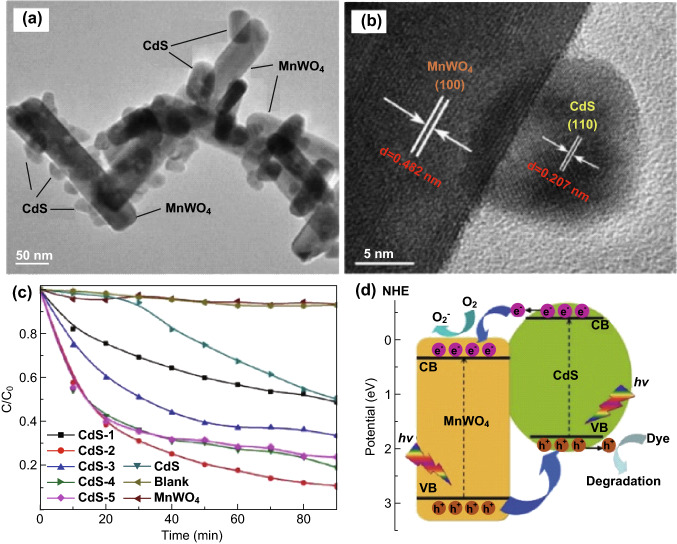
**a**, **b** TEM and HRTEM images of a CdS/MnWO_4_ heterojunction. **c** Photocatalytic degradation of MB over CdS/MnWO_4_ with different CdS contents. **d** Schematic photocatalytic mechanism of CdS/MnWO_4_ heterojunction. Reproduced with permission from Ref. [155]. Copyright 2017 Elsevier

For constructing MWO_4_-based heterojunctions, semiconductors with a narrow band gap were chosen as a counterpart component to form the heterojunction system, which exhibited remarkable advantages for enhancing the photocatalytic performance. Among most semiconductors with a narrow band gap, cadmium sulfide (CdS) has attracted increasing attention for heterojunction fabrication because of its narrow band gap and high CB position, which could be beneficial for photocatalytic H_2_ production [151, 152]. Therefore, various CdS/MWO_4_ heterojunction photocatalysts have been developed, which exhibit enhanced photocatalytic activities in water purification and energy conversion [153, 154]. For instance, Yan et al. [155] prepared a CdS/MnWO_4_ heterojunction using a facile hydrothermal method to mineralize MB and methyl violet (MV) under visible-light irradiation. Owing to the overlapping of energy bands and tightly contacted interfaces between CdS and MnWO_4_, the holes at the VB of MnWO_4_ could transfer to the VB of CdS, and the excited electrons at the CB of CdS could in turn move into the CB of MnWO_4_. This model could efficiently limit the recombination rate of the photogenerated electrons and holes, thus intensifying the separation of the photogenerated charge carriers in the hybrid system (Fig. 14). Nevertheless, it is apparent that the introduced CdS amount was not synchronous with the photocatalytic activities of the heterojunction, while there was an optimal amount of CdS in the hybrid. Excessive CdS amounts caused severe agglomeration of MnWO_4_, damaging the heterojunction and worsening the separation efficiency of the photogenerated electrons and holes. This phenomenon was nearly discovered in the heterojunctions by combining two or more components into one system. In addition, Xu et al. [156] prepared a CdS/ZnWO_4_ heterojunction consisting of ZnWO_4_ NRs and CdS NPs using a hydrothermal method for the photodegradation of ciprofloxacin antibiotics. Compared to the ZnWO_4_ NRs and CdS NPs, the CdS/ZnWO_4_ hybrids showed higher photocatalytic activity than bare ZnWO_4_ NRs and CdS NPs, which was ascribed to the highly efficient separation of the photogenerated electrons and holes in the hybrid structure.

**Fig. 15:**
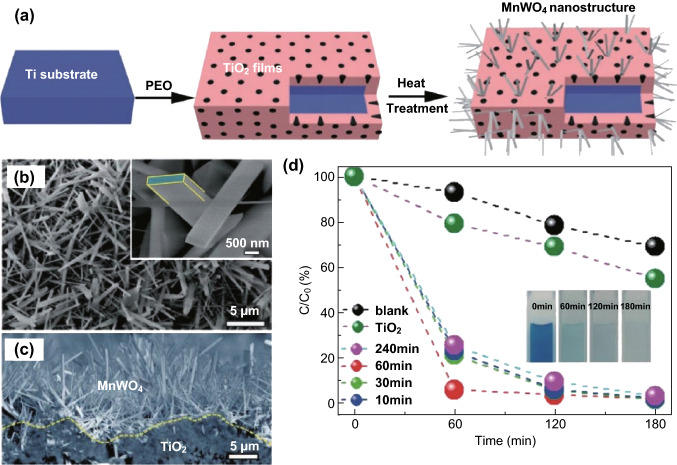
**a** Schematic diagram describing the formation of MnWO_4_ nanoplates. **b–c** FESEM images of the MnWO_4_ nanoplates and MnWO_4_ nanoplates on TiO_2_ film. **d** Photodegradation efficiency of different samples heated at 850 °C for different times. Reproduced with permission from Ref. [162]. Copyright 2016 Royal Society of Chemistry

The CdS/CdWO_4_ heterojunction also exhibited enhanced efficiency in the photocatalytic H_2_ production. For instance, Jia [157] and Wang et al. [158] deposited CdS on the surface of CdWO_4_ via an ion-exchange and in situ growth route, and the fabricated Z-scheme CdS/CdWO_4_ hybrid exhibited significantly enhanced photocatalytic performance for H_2_ evolution compared to that of the pure CdWO_4_ and CdS. As discusses in the previous section, the MWO_4_ with a small M^2+^ cation has a narrow band-gap energy, which can be considered an efficient solar energy harvester to connect with wide band-gap semiconductors for constructing MWO_4_-based heterojunctions [159–161]. Thus, Jiang et al. [162] fabricated an MnWO_4_/TiO_2_ heterojunction with excellent mechanical adhesion by the in situ growth of MnWO_4_ on a porous TiO_2_ film; it presented extremely high photocatalytic performance for degrading MB because of its high crystallinity, large surface area, and strong mechanical properties (Fig. 15). Similarly, Buvaneswari et al. [163] prepared an FeWO_4_/ZnO heterojunction via a simple co-precipitation route; the band gap of the prepared FeWO_4_/ZnO heterojunction was estimated to be 2.12 eV, which was apparently smaller than that of ZnO (3.01 eV). In comparison to pristine ZnO, the photocatalytic activity of the FeWO_4_/ZnO hybrid was substantially enhanced because of the formation of a FeWO_4_/ZnO heterojunction structure. Additionally, the CuWO_4_/ZnO hybrid consisting of ZnO NRs and CuWO_4_ NPs fabricated by Mavric et al. [164] showed enhanced photocatalytic activity compared to that of pure CuWO_4_ NPs and ZnO NRs.

Furthermore, a MO_x_/MWO_4_ hybrid as a smart-built heterojunction was fabricated using a facile one-pot synthetic strategy to enhance the interaction between MO_x_ and MWO_4_, in which M is generally reported to be Fe, Ni, Co, or Cu [165–168]. Cao et al. [169] fabricated a novel p-n heterojunction consisting of Fe_3_O_4_ NPs and FeWO_4_ nanowires. The calculated band gap of the FeWO_4_/Fe_3_O_4_ heterojunction was 2.50 eV, lower than that of pristine FeWO_4_ nanowires. The FeWO_4_/Fe_3_O_4_ heterojunction exhibited enhanced photo-Fenton activity compared to that of the bare FeWO_4_ nanowires under UV–visible-light irradiation with the addition of H_2_O_2_. In addition, a α-SnWO_4_/SnO_2_ heterostructure was synthesized with CTAB as the surfactant [170] and displayed enhanced photocatalytic performance compared to that of pure α-SnWO_4_. Considering that WO_3_ can be obtained by dehydration from tungstate acid, WO_3_ is considered to be simultaneously produced during the synthesis of MWO_4_ and is likely to form a MWO_4_/WO_3_ heterojunction, such as CoWO_4_/WO_3_ [171, 172], NiWO_4_/WO_3_ [173, 174], or CuWO_4_/WO_3_ [175, 176]. Aslam et al. [177] prepared a CdWO_4_/WO_3_ heterojunction using a hydrothermal and chemisorption method, and reported enhanced photocatalytic activities toward the degradation of organic pollutants, compared with pure CdWO_4_ and WO_3_. This section clearly demonstrates in detail that the construction of MWO_4_-based heterojunction systems is an effective and controllable method for enhancing the photocatalytic activities of MWO_4_-based semiconductors.

#### Hybridization with Carbon-Rich Materials

Carbon-rich materials, including carbon nanotubes (CNTs), graphene, and graphitic carbon nitride (g-C_3_N_4_), possess unique physical and chemical properties such as a large surface area, high absorption co-efficiency, and chemical stability, ensuring excellent and long-lasting applications in the fields of photochemical and PEC water treatment, photovoltaic devices, and water splitting [178–183]. Carbon-rich materials have a conjugative π structure and unique *sp*^2^/*sp*^3^ hybrid carbon network, which are suitable substrates for constructing hybrid photocatalysts to intensify the separation and transportation of photoinduced charge carriers inside carbon-rich networks, thus improving the photocatalytic performance [184–187]. Based on this strategy, Gaillard et al. [188] synthesized a novel photoelectrode consisting of CuWO_4_ and a multi-wall CNT (MWCNT) to tune the photogenerated charge transfer in the nanocomposite film for enhancing the performance of solar-driven PEC water splitting. Compared to the bare CuWO_4_ photoelectrode, the resistance and photocurrent density of the CuWO_4_/MWCNT composite photoelectrode decreased and increased by 30% and 26%, respectively. This was mainly attributed to the complete dispersion of the MWCNT as an electron collector in the entire CuWO_4_ layer. Compared to CNTs, graphene nanosheets produced via the chemical oxidation treatment of graphite have more *sp*^3^ hybridized edge structure because of the destroyed perfect *sp*^2^ structure. It is well-known that a perfect *sp*^2^ carbon structure (CNT) is beneficial for rapid charge mobility, and that an *sp*^3^-hybridized carbon structure (graphene) can lead to a small band gap in the semiconductor [189, 190]. Meanwhile, layer-structure carbon materials have a richer porosity substructure assembly from graphene stacking and surface defects, which could provide more reactive sites, in comparison to tube-like carbon materials. Recently, Bai et al. [191] designed a ZnWO_4_/graphene hybrid, demonstrating that graphene could act as a photo-sensitizer in the hybrid and improve the production of ^·^OH and ^·^O_2_^−^ radicals. The excited photogenerated electrons at the CB of ZnWO_4_ could be easily injected into the lowest unoccupied molecular orbital (LUMO) of graphene, resulting in a beneficial spatial separation between the holes and electrons inside the ZnWO_4_/graphene hybrid, in which more holes and electrons can participate in the production of active species, in comparison to bare ZnWO_4_. Xu et al. [192] reported a CdWO_4_/graphene hybrid using a hydrothermal process, in which the formed heterojunction showed significantly enhanced photocatalytic activity compared to that of the bare ZnWO_4_. However, it was found that excessive graphene could have a negative effect on the photocatalytic performance because of the reduced light absorption efficiency of CdWO_4_ with the addition of the superfluous graphene.

**Fig. 16:**
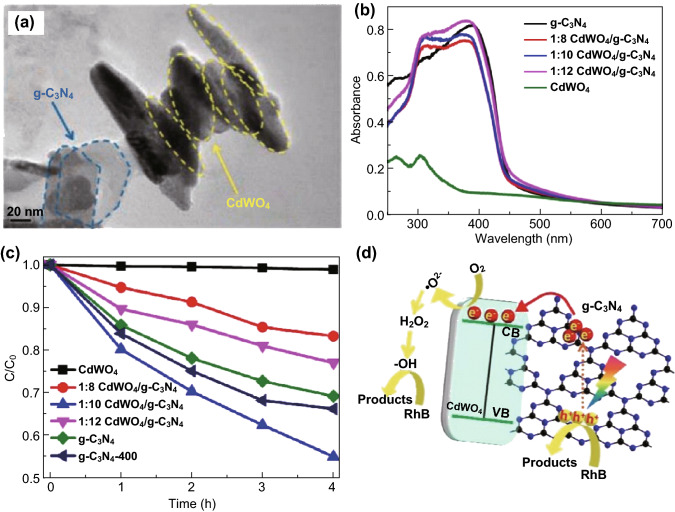
**a** TEM image, **b** UV–vis diffused reflectance spectra, and **c** photocatalytic degradation curves of CdWO_4_/C_3_N_4_. **d** Mechanism of photocatalytic reaction on the CdWO_4_/C_3_N_4_. Reproduced with permission from Ref. [197]. Copyright 2015 Elsevier

Apart from graphene, other carbon-rich materials, such as g-C_3_N_4_ and mesoporous carbon materials, have been considered as promising candidates to build MWO_4_-based heterojunctions. g-C_3_N_4_, which is regarded as an allotrope of C_3_N_4_, possesses excellent chemical stability, a suitable band gap of 2.7 eV, and a high specific surface area, which are beneficial for anchoring various other semiconductor materials [193–195]. For instance, Sun et al. [196] synthesized g-C_3_N_4_/ZnWO_4_ NRs via a thermal treatment route, and investigated the microcosmic mechanisms of the enhanced photocatalytic activity of C_3_N_4_/ZnWO_4_ in comparison to pristine ZnWO_4_ NRs and C_3_N_4_. Together with theoretical calculation results, it was found that the well-matched energy level alignment of the C_3_N_4_ and ZnWO_4_ NRs was the primary reason for the enhancement of the photocatalytic performance, in which the electrons at the VB top edge of the C_3_N_4_, when excited by the incident light, quickly jumped to the CB bottom edge of C_3_N_4_ and then transferred to the CB of ZnWO_4_ because of the staged alignment, thus enhancing the charge transfer and separation efficiency. In addition, Tian et al. [197] synthesized a CdWO_4_/C_3_N_4_ hybrid using a hydrothermal process, followed by a mixed-calcination treatment. Compared to pure CdWO_4_, the CdWO_4_/C_3_N_4_ heterojunction exhibited higher photocatalytic activity (Fig. 16c), the proposed mechanism for which is shown in Fig. 16d. The matched band structures between the two components contributed to the separation and transfer process, in which the excited electrons at the CB of C_3_N_4_ moved to the CB of CdWO_4_ following a downward staged band, such that the photoinduced electrons and holes were efficiently separated, thus enhancing the overall photocatalytic activity. Considering its appropriate band energy level, g-C_3_N_4_ is usually used to construct a Z-scheme hybrid system to enhance photocatalytic activities. Instead of a type-II heterojunction, the direct Z-scheme configuration not only forms a built-in electronic field to promote the separation and transfer efficiency of photogenerated electrons and holes, but it also maintains the reductive and oxidative ability of electrons and holes [198, 199]. Recently, Zhu and co-workers [200] reported a direct Z-scheme heterojunction by combining g-C_3_N_4_ with Ag_2_WO_4_ through a facile precipitation route. Because of the use of g-C_3_N_4_ as a support in the precursor solution, Ag_2_WO_4_ was able to nucleate and grow on the surface of the layered C_3_N_4_, thus resulting in the Ag_2_WO_4_ evenly distributing on the surface of the layered g-C_3_N_4_-nanosheets. In comparison to the bare Ag_2_WO_4_ and g-C_3_N_4_, the Z-scheme of the g-C_3_N_4_/Ag_2_WO_4_ hybrid system exhibited a much higher photoactivity for the degradation of MO, owing to the efficient separation between the photoinduced electrons and holes in the direct Z-scheme configuration. Given the aforementioned examples and explanations, it is obvious that carbon-rich materials possess a high charge carrier mobility and large specific surface area, which could efficiently promote the separation efficiency of photoexcited electrons and holes in the MWO_4_, ultimately leading to improvement of the photocatalytic activity.

## Summary and Outlook

This review summarized the development of novel strategies to enhance the photocatalytic performance of MWO_4_-based materials with a special emphasis on their applications in environmental purification and solar water splitting. Although significant improvements have been achieved in the construction of highly efficient ternary MWO_4_-based oxides, challenges remain that need to be addressed. First, the recombination rate of photogenerated charge carriers for MWO_4_-based photocatalysts is still considerably high, accounting for poor reduction ability in the photoexcited electrons at a low potential of the CB edge, which are easily quenched by defects and holes. Second, although morphological engineering could improve the photocatalytic activity of MWO_4_-based systems by regulating the crystal structure, particle size, and surface area, the current synthetic methods are inadequate for large-scale preparation, particularly for nanosized materials, which would significantly improve the separation efficiency of the photogenerated charge carriers. Third, the surface effect, particularly the crystal surface effect on the photocatalytic performance of MWO_4_-based systems, has not been synergistically and comprehensively investigated. It is thought that the atom configurations and surface defects should be paid more attention, to provide important information for designing highly efficient photocatalysts. Fourth, based on this review, it is clear that the MWO_4_ materials consisting of a single valence metal ion, such as Cd, Zn, or Sn, have been well-developed in the past, while those composed of a versatile valence metal, for instance, Co, Fe, or Ni, have been insufficiently explored in surface engineering and theoretical computation.

To overcome these challenges, future research needs to focus on the exploration of novel photocatalytic materials. Although the sunlight-harvesting ability and separation efficiency of photogenerated charge carriers could be strengthened by heteroatom doping or heterojunction fabrication as reported by previous literature, traditional material screening, high-throughput screening, and computational materials design can guide the construction of photocatalysts with a proper band edge position and suitable band gap, thereby shortening the experimental period, reducing the workload, and saving experimental cost. In other fundamental studies, combining experiment and theory would enable understanding the photocatalytic principles and screen alternative high-performance photocatalysts. Future work also needs to focus on facile synthetic approaches for constructing stable MWO_4_ materials with high active crystal surface and/or quantum size, and developing advanced techniques for large-scale production. It is expected that further progress in ternary MWO_4_-based photocatalysts for applications in environmental purification and solar water splitting will be made in future studies.
